# Sex differences in the effects of aromatherapy on anxiety and salivary oxytocin levels

**DOI:** 10.3389/fendo.2024.1380779

**Published:** 2024-06-11

**Authors:** Daisuke Nakajima, Megumi Yamachi, Shingen Misaka, Kenju Shimomura, Yuko Maejima

**Affiliations:** ^1^Department of Bioregulation and Pharmacological Medicine, Fukushima Medical University School of Medicine, Fukushima City, Fukushima, Japan; ^2^Medical Division, Nitto Boseki Co., Ltd., Koriyama Fukushima, Japan; ^3^Departments of Obesity and Inflammation Research, Fukushima Medical University School of Medicine, Fukushima City, Fukushima, Japan

**Keywords:** aromatherapy, hand massage, oxytocin, anxiety, stress, sex differences

## Abstract

**Objective:**

Aromatherapy is a holistic healing method to promote health and well-being by using natural plant extracts. However, its precise mechanism of action and influence on the endocrine system remains unclear. Since recent studies reported that a neuropeptide, oxytocin, can attenuate anxiety, we hypothesized that if oxytocin secretion is promoted through aromatherapy, it may improve mood and anxiety. The present study is aimed to investigate the relationship between oxytocin and the effects of aromatherapy with lavender oil on anxiety level, by measuring salivary oxytocin levels in healthy men and women.

**Methods:**

We conducted a randomized open crossover trial in 15 men and 10 women. Each participant received a placebo intervention (control group) and aromatherapy with lavender oil (aromatherapy group). For the aromatherapy group, each participant spent a 30-min session in a room with diffused lavender essential oil, followed by a 10-min hand massage using a carrier oil containing lavender oil. Anxiety was assessed using the State-Trait Anxiety Inventory (STAI) before the intervention, 30-min after the start of intervention, and after hand massage, in both groups. Saliva samples were collected at the same time points of the STAI.

**Results:**

In women, either aromatherapy or hand massage was associated with a reduction in anxiety levels, independently. Moreover, salivary oxytocin levels were increased after aromatherapy. On the other hand, in men, anxiety levels were decreased after aromatherapy, as well as after hand massage, regardless of the use of lavender oil. However, there were no significant differences in changes of salivary oxytocin levels between the control and aromatherapy groups during the intervention period. Interestingly, there was a positive correlation between anxiety levels and salivary oxytocin levels before the intervention, but a negative correlation was observed after hand massage with lavender oil.

**Conclusion:**

The results of the present study indicate that in women, aromatherapy with lavender oil attenuated anxiety with increase in oxytocin level in women, whereas in men, there was no clear relationship of aromatherapy with anxiety or oxytocin levels but, there was a change in correlation between anxiety and oxytocin. The results of the present study suggest that the effect of aromatherapy can vary depending on sex.

## 1 Introduction

Aromatherapy is the use of essential oils extracted from plants for the treatment of physical and psychological health. It has been reported to have positive psychological therapeutic effects through inhaling aromatic plant-based compounds (aroma) (1). There are many kinds of aroma oils, and they are used selectively based on the purpose. For example, sweet marjoram aroma has been reported to have calming and sedative effects, and thus contributes to the relief of negative emotional states. On the other hand, lavender aroma has been reported to have positive effects on mood, stress, anxiety, depression and insomnia (1).

Aromatherapy has recently become popular, and is used by a wide range of generations, regardless of gender. The main reason for this popularity is the positive effect that aromatherapy has on mental stress. Stress is considered to play a bigger role in modern society than it ever has before. In particular, stress-related disorders became a global issue as a result of restrictions caused by the COVID-19 pandemic. The global population experiencing mood disorders and depression triggered by stress is increasing, and the incidence of depression is estimated to be 5% in adults, with a higher prevalence in women than in men (2). Therefore, aromatherapy is expected as a simple and effective method to improve mental illness and promote wellness. In addition, massage has been reported to contribute to reduce anxiety, heart rate and stress hormones levels (3). However, in order to apply aromatherapy and massage as possible mental therapies, proper academic evaluation and clarification of their mechanisms of action are necessary.

Oxytocin has been attracting attention as a hormone that attenuates anxiety and stress response (4). It is a peptide hormone consisting of nine amino acids that is secreted from the hypothalamus and acts in both the brain and the body. It was originally discovered as a hormone that promotes delivery and milk ejection in 1906 (5), and has long been regarded as important hormone only during the perinatal period in women. However, it has recently been reported that oxytocin also has functions on behaviors, such as maternal behavior (6), social behavior (7), and social communication regardless of sex (8). Our previous studies found the anti-obesity and anti-metabolic syndrome effects of oxytocin (9, 10). In addition, oxytocin has been reported to be associated with anxiety and stress in animals and humans. Oxytocin exerts a central anxiolytic-like effect on the endocrine system and on behavior (11). Compared to wild-type mice, oxytocin-deficient mice displayed more anxiety-related behaviors and released more corticosterone after experiencing a psychogenic stressor (12). There is a negative correlation between plasma oxytocin level and severity of anxiety in patients with major depression (13). Oxytocin is considered a suitable treatment option for human anxiety disorders, especially for those associated with socio-emotional dysfunctions (14).

Oxytocin is present in breast milk, cerebrospinal fluid, blood, and saliva (15, 16), and it has been reported that salivary and blood oxytocin concentrations increase with massage and/or aromatherapy (17–19). Given these findings, it is speculated that the stress-buffering effects of aromatherapy and/or massage are mediated by oxytocin secretion. Therefore, the present study is aimed to investigate changes in anxiety after aromatherapy and hand massage with aroma oil in men and women, and to elucidate the association with the changes in anxiety and salivary oxytocin levels. To the best of our knowledge, this is the first study to investigate the effects and underlying mechanistic factors of aromatherapy and hand massage by measuring changes in salivary oxytocin levels after these treatments in human men and women.

## 2 Materials and methods

### 2.1 Participants and experimental design

We conducted a 2 × 2 crossover trial in 15 men and 11 women in their 20s–40s. They received prior explanation about the interventions used in this study, and provided their written informed consent to participate in this study. Each participant underwent both interventions in two different days, and to avoid the influence of physiological diurnal variation in oxytocin levels, the interventions were conducted at the same time of day in each intervention. Only one female participant (one out of 25 participants: 4%) had experience of aromatherapy in the past. Thus, almost all participants (96%) have never experienced aromatherapy before and the present study was the first experience. There were no participants who experienced hand massage in the past. The interval between the two experimental days was set to be at least one week. The participants did not include smokers, individuals with chronic illnesses, those taking medication (including traditional Chinese medicine), or those who were pregnant or lactating.

### 2.2 Intervention protocol

**Figure 1:**
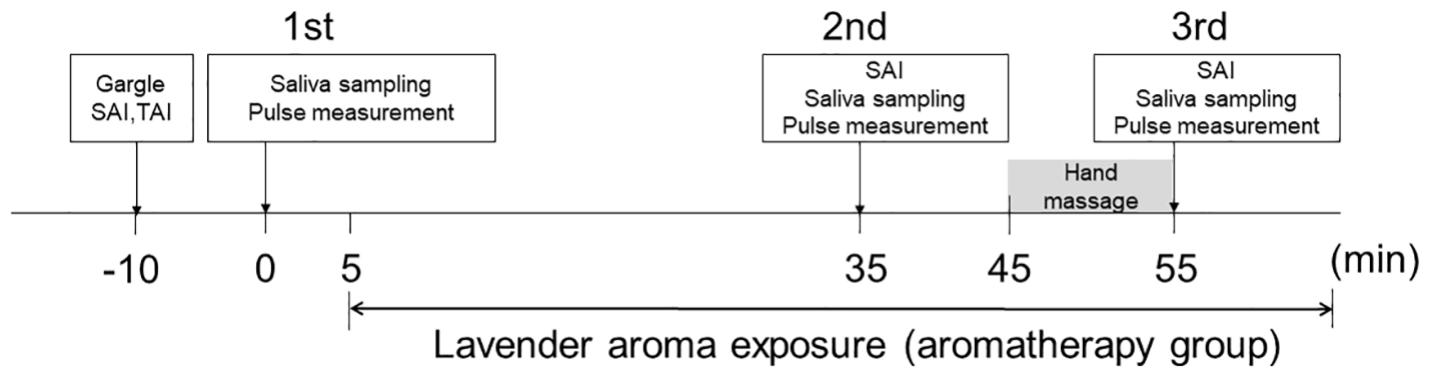
Protocol for interventions. The participants received two interventions (aromatherapy and control). Sample collection, SAI and TAI questionnaires, and pulse measurement were performed three times during each intervention (1st assessment, 2nd assessment, 3rd assessment). Prior to each intervention, the participants underwent the 1st assessment. They then spent 30-min in a room with diffused aroma (aromatherapy group) or without (control group), followed by the 2nd assessment. Saliva sampling, completing SAI score and pulse measurement were performed within 10 min (35-45 min). They subsequently received a 10-min hand massage with aroma oil (aromatherapy group) or without (control group), followed by the 3rd assessment.

Anxiety was assessed using the State-Trait Anxiety Inventory (STAI) composed of State Anxiety Inventory (SAI) and Trait Anxiety Inventory (TAI) questionnaires freely available for academic use (20, 21) and the reference reported by Shimizu and Imae (22). SAI was evaluated for each intervention since it reflects current emotional state and ideal to evaluate time dependent change of emotion, while TAI was measured to evaluate general mood. Saliva samples were collected prior to intervention, and the participants were asked to complete the SAI and TAI questionnaires (1st assessment). Each participant subsequently received a placebo intervention (control group) or aromatherapy intervention (aromatherapy group). The participants in the aromatherapy group received a 30-min aromatherapy session in a private room with diffused aroma oil, as described in 2.3. During the session, they were prohibited from doing anything that could affect their anxiety state, such as using a smartphone, reading, or sleeping. After the session, saliva samples were collected, and the participants were asked to complete the SAI questionnaire again, and measure the heart rate (2nd assessment). This saliva sampling, completing SAI score questionnaire and pulse measurement were performed within 10 min (35-45 min in **Figure 1**). They then received a 10-min hand massage with aroma oil in the same room, followed by sample collection and the SAI questionnaire (3rd assessment). The participants in the control group stayed in a private room without diffused aroma oil for 30-min after the 1st assessment. Following the 2nd assessment, they received a 10-min hand massage using carrier oil without containing aroma oil, followed by the 3rd assessment. As described above, SAI evaluates current emotional state and TAI measures trait emotional mood. Thus, TAI test was performed only at the 1st assessment. The detailed protocol is illustrated in **Figure 1**.

### 2.3 Aromatherapy and hand massage

Aromatherapy was administered using authentic lavender essential oil (*Lavandula angustifolia*: from France, TREE OF LIFE Co., Ltd.), which was introduced into the room using a diffuser (Brezza Corporation Flavor Life, Tokyo, Japan). For hand massage, sweet almond oil (TREE OF LIFE Co., Ltd.) was used as the carrier oil. In the aromatherapy group, lavender oil concentration of 1% (v/v) was added to the carrier oil. All hand massages were administered for 10-min (5-min on each hand) by a nurse trained in lymphatic massage. In control intervention, the participants were not given both moisture diffuser and aroma oil and were asked to take rest in intervention room for 30 min. The participants received hand massage with almond oil without lavender essential oil in control intervention.

To ensure consistency in the interventional conditions, all hand massages were performed by the same nurse.

### 2.4 Measurement of heart rate

Heart rate was measured for 30-sec with the participant in a sitting position, using a pulse wave analyzer with a fingertip sensor (Act Medical Service Co., Fukushima, Japan). This measurement was performed 5-min after each of the 1st, 2nd, and 3rd saliva samplings (**Figure 1**).

### 2.5 Saliva collection and storage

Saliva was collected using a Salivette® tube (Sarstedt AG, Nümbrecht, Germany). In order to prevent degradation of oxytocin, aprotinin (600 KIU; 014-18113, FUJIFILM Tokyo, Japan) was added to the tube. The participants were not allowed to eat or drink anything except water from 2-h prior to the intervention. They rinsed their mouths thoroughly with water 10-min prior to the first saliva collection. Although drinking water was allowed during the intervention, it was prohibited for 10-min prior to each saliva collection. Samples were centrifuged (1000 × *g* for 5-min) after collection, and the supernatant was collected and stored at -80°C until the day of measurement.

### 2.6 Measurement of salivary oxytocin

At the measurements, 0.1 M HCl was added to the supernatant, and oxytocin was recovered by a C18 Sep-Pak column (Waters Co., Massachusetts, U.S.A.) and was extracted with methanol. Salivary oxytocin was measured using an ELISA kit (K048-H, ARBOR Assays Inc., Michigan, U.S.A). In order to minimize inter-measurement variation, samples from each participant were measured using the same assay plate. GraphPad Prism 7.0 (Dotmatics Co., Massachusetts, U.S.A.) was used for statistical analysis.

### 2.7 Statistical analysis

All data are presented as mean ± standard error. Comparison of the TAI and SAI scores between the control and aromatherapy groups was conducted using a paired *t*-test. Pearson’s correlation coefficient was used to determine the correlation between SAI and TAI scores, and Spearman’s rank correlation coefficient was used to analyze the correlation between SAI score and oxytocin concentration. Heart rate, SAI score, and oxytocin concentration at the 1st, 2nd, and 3rd assessments in the control and aromatherapy groups were analyzed by one-way ANOVA. Two-way ANOVA was used to compare the percentage of oxytocin concentrations, which were set as 100% at the 1st sampling, between the control and aromatherapy groups. All statistical tests were two-tailed, with *p* values of < 0.05 considered statistically significant.

## 3 Results

### 3.1 Participants

**Figure 2:**
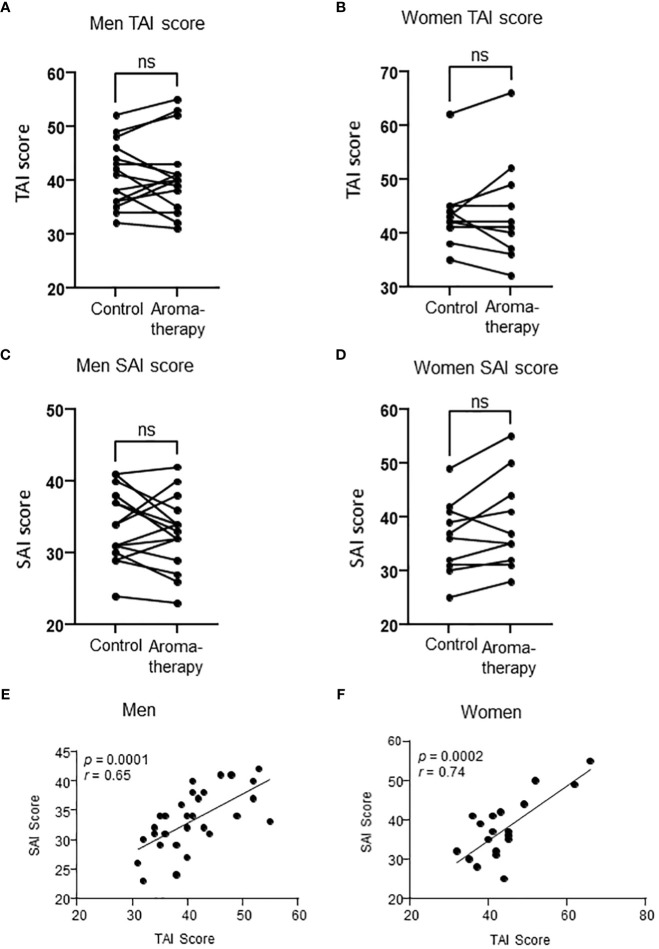
Relationship between TAI score and SAI score in men and women. **(A, B)** TAI scores in the control and aromatherapy groups in men (**A**, *n* = 15) and women (**B**, *n* = 10). ns: *p* > 0.05, paired t-test. **(C, D)** SAI score at the 1st assessment in the control and aromatherapy groups in men (**C**, *n* = 15) and women **(D)**, *n* = 10). ns: *p* > 0.05, paired *t*-test. **(E, F)** The correlation of SAI score and TAI score at the 1st assessment in the control and aromatherapy groups in men (**E**, *n* = 30) and women (**F**, *n* = 20). **(E, F)** analyzed by Pearson’s correlation coefficient. ns, not significant.

Fifteen men and 11 women participated in this study. One of the female participants was excluded from the analysis because the amount of saliva collected was not enough for assay. The mean age of the participants included in the analysis was 24.9 ± 0.8 years (21–32 years) and 26.5 ± 2.7 years (20–42 years) in the men and women, respectively. The mean BMI was 21.5 ± 0.7 (17.3–26.5) in the men, and that in the women was 22.3 ± 0.7 (20.0–27.4). The mean TAI scores in the control and aromatherapy groups were 40.9 ± 1.9 (31–55) and 44.0 ± 3.1 (32–66) in the men and women, respectively. The mean SAI score at before the intervention in control and aromatherapy groups were 32.7 ± 1.2 (23–41) and 36.8 ± 2.6 (25–55) in men and women, respectively. The mean salivary oxytocin levels before the intervention in the control and aromatherapy groups were 68.3 ± 19 (17.3-294) and 66.5 ± 8.3 (25-98.5) in men and women, respectively. There were no significant differences in SAI score and oxytocin levels before the intervention between men and women (**Table 1**). There were no significant differences in TAI or SAI scores between the control and aromatherapy groups (**Figures 2A–D**). Additionally, there was a significant positive correlation between the TAI and SAI scores at the 1st assessment in both men and women (*r* = 0.65, *p* < 0.001; *r* = 0.74, *p* < 0.001; respectively **Figures 2E, F**).

**Table 1:** Participant characteristics.

	Men (*n* = 15)			Women (*n* = 10)			p-value
	Mean	SE	Range	Mean	SE	Range	
**Age (years)**	24.9	0.8	21-32	26.5	2.7	20-42	0.5246
**Height (cm)**	173.1	1.7	163-184	160.0	1.9	154-168	< 0.0001
**Weight (kg)**	64.5	2.4	50-84	57.1	2.0	51-71	< 0.05
**BMI (kg/m²)**	21.5	0.7	17.3-26.5	22.3	0.7	20.0-27.4	0.4221
**TAI**	40.5	1.7	31-55	43.6	2.8	32-66	0.3226
**SAI 1st**	32.7	1.2	23-41	36.8	2.6	25-55	0.1221
**Oxytocin 1st (pg/ml)**	68.3	19	17.3-294	66.5	8.3	25-98.5	0.9431

### 3.2 Relationships among aromatherapy, hand massage and SAI score

#### 3.2.1 Aromatherapy with lavender oil had no effect on SAI scores in men

**Figure 3:**
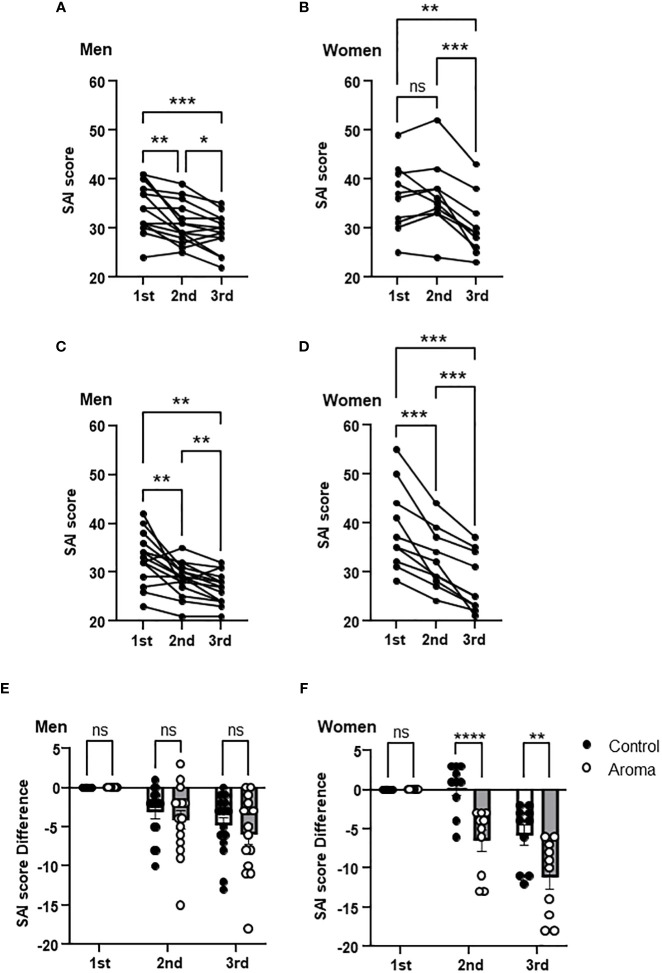
Change of SAI scores in the control and aromatherapy groups. **(A, B)** Change of SAI scores in the control group in men (**A**, *n* = 15) and women (**B**, *n* = 10). One-way ANOVA Holm-Sidak’s multiple comparisons test. (**C, D)** Change of SAI scores in the aromatherapy group in men (**C**, *n* = 15) and women (**D**, *n* = 10). One-way ANOVA Holm-Sidak’s multiple comparisons test. (**E, F)** Comparison of SAI score difference from 1st assessment at each assessment between the control and aromatherapy groups in men (**E**, *n* = 15) and women (**F**, *n* = 10). Two-way ANOVA followed by Sidak’s multiple comparisons test. ns, *p* > 0.05; *p < 0.05; **p < 0.005; ***p < 0.0005; ****p < 0.0001. ns, not significant.

A significant decrease was observed in SAI scores at the 2nd and 3rd assessments in the men of both the control and aromatherapy groups. (**Figures 3A, C**). To evaluate the effect of each intervention on SAI scores, changes in the SAI scores from the 1st assessment [ΔSAI scores: 2nd score – 1st score (2nd point), 3rd score-1st score (3rd point)] were compared between the control and aromatherapy groups. There was no significant difference in ΔSAI score between the control (-3.1 ± 0.86) and aromatherapy (-4.1 ± 1.2) groups at the 2nd assessment. Similarly, there was also no significant difference in ΔSAI score between the control (-4.8 ± 1.0) and aromatherapy (-5.9 ± 1.3) groups at the 3rd assessment (*F_1,28 = _* 0.5057, *p* = 0.48, **Figure 3E**).

#### 3.2.2 Aromatherapy with lavender oil and hand massage decreased SAI score in women

The SAI scores at the 2nd assessment were significantly decreased in women in the aromatherapy group (**Figure 3D**), whereas no significant changes were observed in the control group (**Figure 3B**). On the other hand, the SAI scores at the 3rd assessment were significantly decreased in both groups (**Figures 3B, D**). There was a significant difference in the ΔSAI score: score at the 2nd assessment between the control (-0.3 ± 1.0) and aromatherapy (-6.5 ± 1.3) groups (**Figure 3F**). The ΔSAI score at the 3rd assessment was significantly lower in the aromatherapy group (-11.2 ± 1.5) compared with the control group (-5.8 ± 1.3) (*F_1,18 = _* 12.87, *p* = 0.0021, **Figure 3F**).

### 3.3 Heart rate

**Figure 4:**
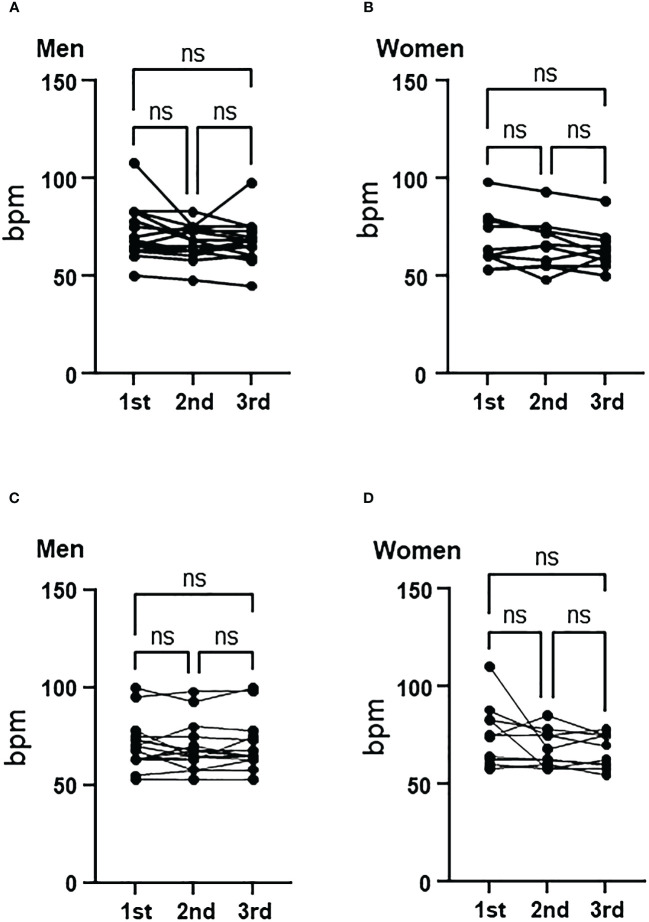
Change of heart rate in the control and aromatherapy groups. **(A, B)** Change of heart rate in the control group in men (**A**, *n* = 15) and women (**B**, *n* = 10). Related-samples Friedman’s ANOVA test. **(C, D)** Change of heart rate in the aroma group in men (**C**, *n* = 15) and women (**D**, *n* = 10). Related-samples Friedman’s ANOVA test. ns, not significant.

There were no significant changes in heart rate in both men and women in the control and aromatherapy groups (**Figure 4**).

### 3.4 Relationships among aromatherapy, hand massage and salivary oxytocin

#### 3.4.1 Salivary oxytocin levels were not changed by aromatherapy and hand massage in men

**Figure 5:**
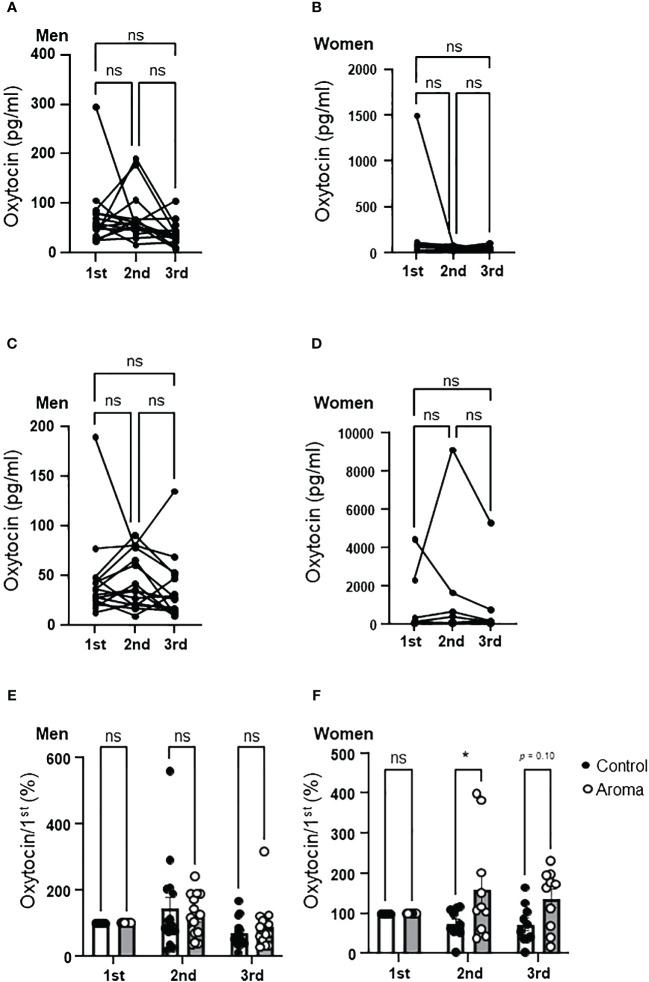
Change of salivary oxytocin levels in the control and aromatherapy groups. **(A, B)** Change of salivary oxytocin concentration (pg/ml) in the control group in men (**A**, *n* = 15) and women (**B**, *n* = 10). Related-samples Friedman’s ANOVA test. **(C, D)** Change of salivary oxytocin concentration (pg/ml) in the aroma group in men (**C**, *n* = 15) and women (**D**, *n* = 10). Related-samples Friedman’s ANOVA test. **(E, F)** Comparison of change of salivary oxytocin levels at each assessment between the control and aromatherapy groups in men (**E**, *n* = 15) and women (**F**, *n* = 10). The oxytocin levels at the 1st assessment is normalized as 100%. Two-way ANOVA followed by Sidak’s multiple comparisons test. ns, *p* > 0.05; * *p* < 0.05. ns, not significant.

There were no significant changes in salivary oxytocin levels from the 1st to the 3rd assessment in men of either the control or aromatherapy group (**Figures 5A, C**). As shown in **Figures 5A, C**, the salivary oxytocin levels largely varied among individuals. Thus, we analyzed the individual changes by setting the salivary oxytocin levels at the 1st assessment as 100%. As shown in **Figure 5E**, no significant differences were found at any points between the aromatherapy group (2nd, 145 ± 36%; 3rd, 70 ± 11%) and the control group (2nd, 121 ± 17%; 3rd, 91 ± 18%) (*F_1, 28_ = *0.0058, *p* = 0.71).

#### 3.4.2 Salivary oxytocin levels were increased by aromatherapy in women

Although an increasing trend was observed in salivary oxytocin levels at the 2nd assessment in women of the aromatherapy group, there were no significant differences between the control and aromatherapy groups at any assessment point (**Figures 5B, D**). As in the case with men, we analyzed the individual changes by setting the salivary oxytocin levels at the 1st assessment as 100%, which showed a significantly higher percent increase at the 2nd assessment in the aromatherapy group (160 ± 42%) than the control group (75 ± 11%) (*F_1, 1_*_8_* = *7.8, *p* = 0.017, **Figure 5F**). Although there was no statistically significant difference in percent change of oxytocin levels between the control and aroma groups, the tendency of increased salivary oxytocin percentage was maintained after the hand massage (3rd point; 73 ± 15% in the control group; 137 ± 23% in the aromatherapy group, *p* = 0.0997).

### 3.5 Relationships among anxiety and salivary oxytocin levels

#### 3.5.1 Effect of hand massage with aroma oil on correlation between anxiety and oxytocin levels in men

**Figure 6:**
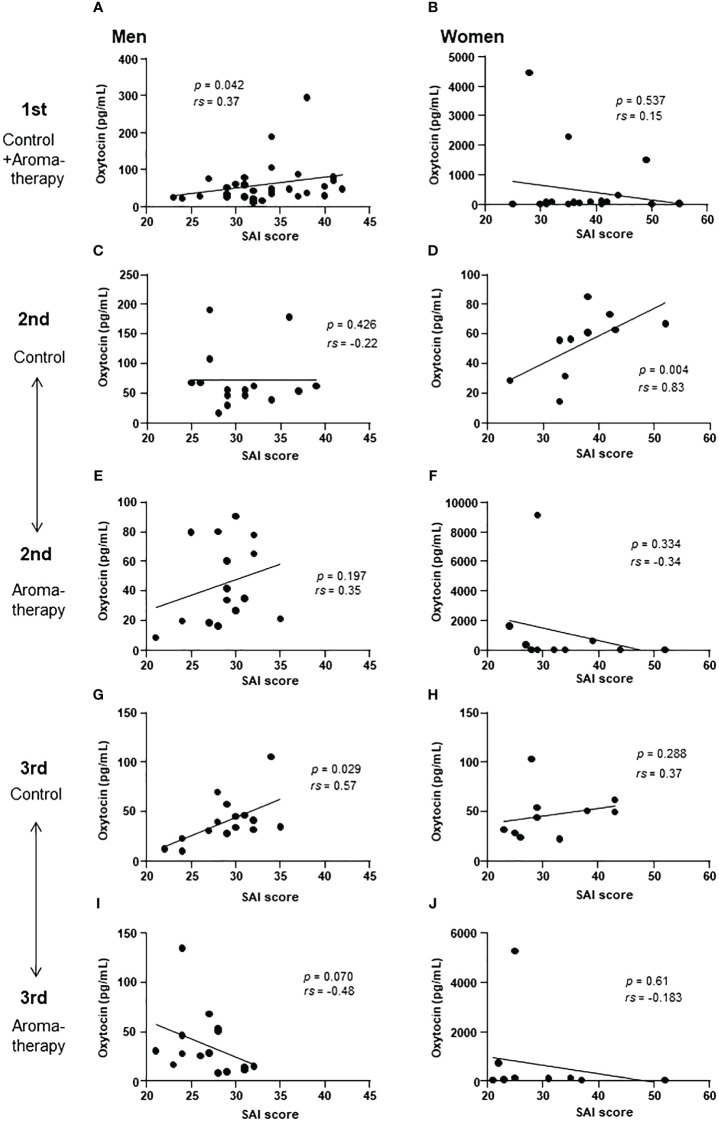
Correlation of salivary oxytocin level and SAI score at each assessment. **(A, B)** Correlation of salivary oxytocin levels and SAI scores at the 1st assessment in the control and aromatherapy groups in men (**A**, *n* = 30) and women (**B**, *n* = 20). **(C, D)** Correlation of salivary oxytocin levels and SAI scores at the 2nd assessment in the control group in men (**C**, *n* = 15) and women (**D**, *n* = 10). **(E, F)** Correlation of salivary oxytocin levels and SAI scores at the 2nd assessment in the aromatherapy group in men (**E**, *n* = 15) and women (**F**, *n* = 10). **(G, H)** Correlation of salivary oxytocin levels and SAI scores at the 3rd assessment in the control group in men (**G**, *n* = 15) and women (**H**, *n* = 10). **(I, J)** Correlation of salivary oxytocin levels and SAI scores at the 3rd assessment in the aromatherapy group in men (**I**, *n* = 15) and women (**J**, *n* = 10). **(A–J)** were analyzed using Spearman’s rank correlation coefficient.

The correlations between salivary oxytocin levels and SAI scores at each assessment were analyzed in the control (**Figures 6A, C, G**) and aromatherapy groups (**Figures 6A, E, I**). In men, a significant positive correlation was found at the 1st and 3rd assessments in the control group (**Figures 6A, G**) However, a tendency of negative correlation was observed at the 3rd assessment in the aromatherapy group (**Figure 6I**).

#### 3.5.2 The relationship between SAI score and salivary oxytocin levels in women

In women, there was no significant correlation between the SAI scores and salivary oxytocin level at the 1st assessment (**Figure 6B**). Although a correlation was detected at the 2nd assessment in the control group (**Figure 6D**), there were no significant correlations between SAI score and oxytocin level at the 3rd assessment point in both the control and aromatherapy groups (**Figures 6H, J**, respectively).

## 4 Discussion

Many studies have reported the positive effect of aromatherapy on anxiety reduction (23–31). Animal studies in the past have indicated that aromatherapy can provide psychological effects by acting on the central nervous system (32, 33).

In the present study, we examined the changes in anxiety and salivary oxytocin levels induced by aromatherapy and hand massage with aroma oil in individuals aged 20s–40s, including both men and women.

We used the STAI to evaluate the effect of aromatherapy and hand massage on anxiety levels. Previous studies have reported that aromatherapy with lavender oil significantly reduced anxiety in male as well as in female (23–29, 34). In the present study, the SAI scores decreased in both the control and the aromatherapy groups in men, and when considering only men with high initial anxiety (SAI score > 36), a decrease in scores by ≥ 5 points was observed in 50% (3 out of 6) in the control group and 100% (4 out of 4) in the aromatherapy group. These results suggest that although there is no effect in salivary oxytocin levels, aromatherapy on its own may be useful in reducing anxiety in men with high anxiety levels. Further study is required to clarify this effect and its mechanisms.

The present study showed significant differences between the aromatherapy and control groups in SAI score changes from the 1st to 2nd assessment among women. Although there were no significant changes in the SAI scores from the 1st and 2nd assessment in the control group (**Figure 3B**), a significant decrease was observed in the aromatherapy group (**Figure 3D**, 3F). These data suggest that, in contrast to men, simply resting in a quiet room alone does not significantly alter anxiety levels in women. However, exposure to lavender oil appears to have a pronounced anxiety-reducing effect in women. In addition, our results suggest that this reduction in anxiety may be further enhanced by the addition of hand massage.

We measured salivary oxytocin levels based on the hypothesis that the anxiety-reducing effects of aromatherapy and/or massages are mediated by oxytocin secretion. Oxytocin is mainly synthesized in the paraventricular and supraoptic nuclei in the hypothalamus. Oxytocin in the peripheral circulations is derived from oxytocin neurons in magnocellular subdivisions in the paraventricular nucleus and supraoptic nuclei that project to the posterior pituitary. On the other hand, oxytocin neurons in the parvocellular subdivision of paraventricular nucleus secrete oxytocin into the brain or cerebrospinal fluid (CSF) via their projection or dendritic release (35). The level of oxytocin in saliva is considered to reflect that in the CSF (36). Thus, the change of salivary oxytocin levels shown in the present study may reflect the change of oxytocin levels in the CSF. The secretion of oxytocin in the brain plays an important role in reducing stress responses (37). Upon stress, corticotropin-releasing hormone from the paraventricular nucleus of the hypothalamus stimulates the secretion of adrenocorticotropic hormone from the pituitary gland, and adrenocorticotropic hormone stimulates cortisol secretion from the adrenal gland. This axis, known as the hypothalamic-pituitary adrenal (HPA) axis, plays an important role in protecting animals, including humans, from stress. In response to activation of the HPA axis, oxytocin inhibits the expression and secretion of corticotropin-releasing hormone from the hypothalamic paraventricular nucleus, and prevents overactivation of the HPA axis, resulting in attenuation of stress response (37). Therefore, increase in salivary oxytocin level can be considered as an indicator for stress response attenuation.

A significant positive correlation was detected between the SAI scores and salivary oxytocin levels at the 1st assessment in men (**Figure 6A**). This result indicates that the participants with higher anxiety scores showed higher salivary oxytocin levels. Considering the role of oxytocin in stress response, its secretion could be increased to attenuate anxiety. In humans, oxytocin levels in saliva and peripheral blood increase due to factors such psychological stress, exercise, and noise stress (38–41). These findings are consistent with our results found in men, which showed a positive correlation between the SAI scores and salivary oxytocin levels. However, the positive correlation between the SAI scores and salivary oxytocin levels was maintained to some extent at the 3rd assessment in men of the control group (**Figure 6G**). In contrast, there was a tendency of negative correlation at the 3rd assessment in the aromatherapy group (**Figure 6I**). The physiological role of this invert from positive correlation to negative correlation remains unknown. Further study is required to clarify the link between anxiety attenuation and its involvement of oxytocin.

One possible mechanism of oxytocin to attenuate stress is its effect on the amygdala (42, 43). The amygdala is a region of the cerebral limbic system that governs emotions, especially anxiety. Oxytocin receptors are abundantly expressed in this region (44). It has been reported that there is a positive correlation between salivary oxytocin levels and the size of the amygdala in men (45). Furthermore, nasal oxytocin administration attenuates the activation of the amygdala’s response to fearful facial expressions in men (46). In addition, oxytocin can modulate amygdala reactivity in response to social threat and attenuate anxiety (42). Thus, it is possible that higher oxytocin levels contributed to attenuation of anxiety in men with higher anxiety scores in the present study.

Interestingly, contrary to men, there is a negative correlation between salivary oxytocin levels and amygdala volume in women (45). Shou et al. speculated that high oxytocin levels increase amygdala volume by activating this brain region with a high frequency in men, while in women it reduces amygdala volume by suppressing its activation (45). Taking into account this report and our results, it is possible that there is a sex difference in the mechanism of action of oxytocin on anxiety reduction in humans.

Although present study is based on small number of participants, it demonstrated a possible sex difference in the effect of aromatherapy on salivary oxytocin levels. There were no significant differences in the changes of salivary oxytocin levels between the control and aromatherapy groups in the men (**Figure 5E**), while the change of salivary oxytocin levels of the aromatherapy group was significantly larger than that of the control group in the women (**Figure 5F**). In the present study, salivary oxytocin levels at the 2nd assessment in female aromatherapy group increased by approximately 1.5 times differ from to those of the control group, which is a similar level of increase in salivary oxytocin reported in a previous study which postmenopausal women were exposed to lavender essential oil for 20-min (19). The female participant of present study is all premenopausal women and our results suggest that the effectiveness of aromatherapy for anxiety reduction with possible interaction with oxytocin secretion is limited to women.

Although many mechanisms can be speculated for its effectiveness on women, one possible mechanism can be the differences in the olfactory system. The sense of smell in women has been reported to be more sensitive than in men (47). The olfactory bulb is an initial brain region that receives olfactory stimuli from olfactory receptors. A previous postmortem brain study reported that the number of neurons and glial cells in the olfactory bulb of women were 40%–50% higher than those in men (48). Given these findings, the sex difference in the response to aromatherapy may be linked to the sex differences in the olfactory system.

Regarding the levels of oxytocin with the sex difference in the response to aromatherapy, the neurons in olfactory bulb which project to various brain regions related to the stress responses, including hypothalamus, bed nucleus of the stria terminalis and medial amygdala (49–51), may contribute to the results shown in this study. Since, the number of neurons and glial cells in the olfactory bulb of women are higher than those of men (48), the input from olfactory neurons in women may have stronger effects of aromatic effects on enhancing oxytocin response. On the other hand, it has been reported that lavender essential oil inhalation to mice decreased an anxiolytic-like behavior via serotonergic neuronal system (52). Oxytocin increases the release of serotonin and availability of 5-HT1A receptors (53). In addition to these reports, estrogen increases oxytocin receptors expressions in neurons (54).

Considering from these reports, olfactory systems in female can enhance aromatherapy effects via neuronal projection from olfactory neurons than male. In addition, serotonin and estrogen, which have important role in regulating physiology of women, are also known to enhance the oxytocin effect. It is possible to consider that in addition to enhanced olfactory systems, serotonin and estrogen systems further enhanced the role of oxytocin in response to aromatherapy in decreasing anxiety.

Interestingly, in the present study, an additive effect on the increase in salivary oxytocin levels by hand massage was not observed in either sex. According to previous studies, salivary oxytocin levels increase with stroking head (55). On the other hand, oxytocin levels in the blood increase with massage on the back and feet (18, 56). The secretion of oxytocin into saliva and blood depends on the type of stimulus and does not always occur in parallel (57); therefore, it is plausible that the hand massage in the present study did not contribute to an increase in salivary oxytocin levels. Further investigation into this is required.

### 4.1 Limitations

Oxytocin has been reported to be secreted in response to stress, and its secretion in the central and peripheral regions is not necessarily consistent and depends largely on the type of stress (57). Therefore, in the present study, we measured oxytocin level in saliva but not in blood. In addition, given that the oxytocin levels in premenopausal women fluctuate according to the estrous cycle, the correlation between the SAI scores and oxytocin levels in women of the present study may have been interfered by factors other than anxiety. Also, present study is based on relatively small number of participants and further study with larger scale is required.

### 4.2 Conclusion

The results of the present study suggest that aromatherapy with lavender oil attenuated anxiety with increase in oxytocin secretion in women but not in men. This indicates the possibility that attenuation of anxiety by aromatherapy with lavender oil in women may partially depend on oxytocin secretion. However, further research is needed to determine the significant roles of oxytocin in change of anxiety score by aromatherapy and hand massage in men with high anxiety score.

## Data availability statement

The raw data supporting the conclusions of this article will be made available by the authors, without undue reservation.

## Ethics statement

The studies involving humans were approved by General Ethics Committee of Fukushima Medical University. The studies were conducted in accordance with the local legislation and institutional requirements. The participants provided their written informed consent to participate in this study.

## Author contributions

DN: Formal analysis, Writing – original draft, Writing – review & editing. MY: Investigation, Writing – review & editing. SM: Methodology, Supervision, Writing – review & editing. KS: Investigation, Methodology, Supervision, Writing – original draft, Writing – review & editing. YM: Funding acquisition, Investigation, Methodology, Project administration, Supervision, Writing – original draft, Writing – review & editing.
